# New Structural Economic Analysis of Anti-COVID-19 Pandemic Model of BEST Region

**DOI:** 10.3390/ijerph18157822

**Published:** 2021-07-23

**Authors:** Fang Wu, Qi Hu, Chenming Zhu, Haitao Wang, Qian Yu, Huaping Sun

**Affiliations:** 1 School of International Pharmaceutical Business, China Pharmaceutical University, Nanjing 211198, China; 1020112190@cpu.edu.cn (F.W.); huqi15026837306@163.com (Q.H.); zcm18795993365@163.com (C.Z.); wht19971121@163.com (H.W.); yu1518144047@126.com (Q.Y.); 2 School of Finance & Economics, Jiangsu University, Zhenjiang 212013, China

**Keywords:** anti-COVID-19 pandemic method, facilitating state, efficient market, capable organization, new structural economics

## Abstract

The successful anti-COVID-19 pandemic model of BEST region (Beijing-Seoul-Tokyo) includes China, Japan and South Korea, which benefit from its well-functioning organizational ecosystem and specific anti-COVID-19 pandemic strategies. Under the premise of an efficient market, the capable organizations of China, Japan and South Korea will play the dynamic function of coordination and organic connection. They will also help improve the governance efficiency of facilitating state in different stages of fighting against the pandemic. This article follows the analytical logic of the new structural economics, taking the factor endowment and its structure as the starting point for the analysis, through the comparative advantage operation mode determined by the market, and based on the collaborative anti-COVID-19 pandemic perspective of the government, the market and various social organizations, to build a framework for the facilitating state-efficient market-capable organization. The key to the success of the anti-COVID-19 pandemic method in China, Japan and South Korea is organically coordinated between government, market and organizations. Based on the effective promotion of micro-organizations, governments organize resource integration and implement macro-control of the market. A dynamic balance between economic governance and pandemic prevention and control has been achieved by optimizing the endowment structure of resources, improving infrastructure and reducing system costs.

## 1. Introduction

With the outbreak of Coronavirus disease (COVID-19) pandemic in early 2020, the world is rapidly forming a common anti-COVID-19 pandemic stance, and the anti-COVID-19 pandemic cooperation of neighboring countries or regions is essential. China, Japan and South Korea are located in East Asia and have close exchanges between countries. These three countries have more than 30 years of cooperation experience in medical and health care. In 1989, BEST areas, including China, Japan and South Korea, jointly established an academic conference to discuss the epidemiological trend, treatment model and development of colorectal cancer in the three countries. After the SARS outbreak in 2003, the three countries held an international seminar on SARS prevention and control in Beijing. As early as the SARS pandemic, East Asian countries began to cooperate to deal with infectious diseases [1]. Subsequently, to further promote exchanges and cooperation in public health, China, Japan and South Korea held the first China-Japan-Korea Health Ministers Meeting to deal with infectious diseases in 2007. This has been held for twelve consecutive sessions, with many years of cooperation experience and results, reflecting the necessity of cooperation among East Asian countries to a certain extent.

During the COVID-19 pandemic, countries worldwide have participated in a low degree of joint anti-pandemic efforts by regional economies, and there are few relevant studies on this issue. The Regional Comprehensive Economic Partnership and the Comprehensive and Progressive Agreement for Trans-Pacific Partnership are constantly reshaping the cooperation pattern in East Asia [2]. Facing the COVID-19 pandemic, China, Japan and South Korea also responded quickly, utilizing the prevention and control method to minimize the pandemic, and these joint anti-COVID-19 pandemic results have attracted much attention. The China-Japan-Korea Special Foreign Ministers’ Meeting and the Health Ministers’ Meeting have repeatedly mentioned strengthening the joint prevention and control mechanisms of the COVID-19-pandemic, and intensifying the communication and technical cooperation of the three countries’ medical information, which has attracted many scholars to study the feasibility and possible effectiveness of pandemic prevention cooperation. Han Xiandong and Xue Meifang conducted theoretical research on the cooperation methods between China, Japan and South Korea [3]. Wang Junsheng and Tian Derong looked at how to promote practical cooperation between the three countries [4]. Ge Jianhua and Ma Lan reviewed the exchanges and cooperation between the three countries during the pandemic and summarized the unique “East Asian anti-pandemic” model [5]. However, the existing articles mostly examine the anti-COVID-19 pandemic method of China, Japan and South Korea from the perspective of cooperation between the governments of the three countries, and few have done comprehensive analysis and research on the emergency management and anti-COVID-19 pandemic results of public health emergencies in China, Japan and South Korea. This article introduces the emergency management systems of China, Japan and South Korea and the specific measures taken by the three countries in this fight against the pandemic. This is based on the analysis framework of “facilitating state, efficient market and capable organization” in the new structural economics, analyzing the role played by the three main bodies in the countries’ fight against the COVID-19 pandemic.

## 2. “Three Elements” Analytical Frameworks of New Structural Economics

Regarding the role of government or state in economic governance, there have been long-term disputes in economics. For instance, in the famous dispute between Smith, Pigou and Coase, where Smith believed that the government should insist on being the “night watchman” of the economic system, Pigou believed that the government’s taxation could reduce the negative externalities of economic behavior and Coase believed that externalities could be resolved by private negotiation. The new structural economics’ analysis framework emphasizes the construction of an efficient market to promote a facilitating state. China’s economic development secret is the facilitating state, and Professor Lin Yifu first proposed the efficient market. If the market is efficient, the richness of a country’s production factor resources can be fully reflected in the price. Facilitating state can take advantage of the situation, deal with the externalities of innovators, give full play to collaborative governance capabilities [6,7] and improve hardware and software infrastructure to match new industries and technologies [8]. Professor Lin believed that developing countries in transition should play the decisive role of the market in allocating resources, and the government should use macro-control measures to prevent market failure. An efficient market and a facilitating state are the elements of economic development proposed by Professor Lin, and a summary of the experience of economic development and transformation with Chinese characteristics [9].

**Figure 1:**
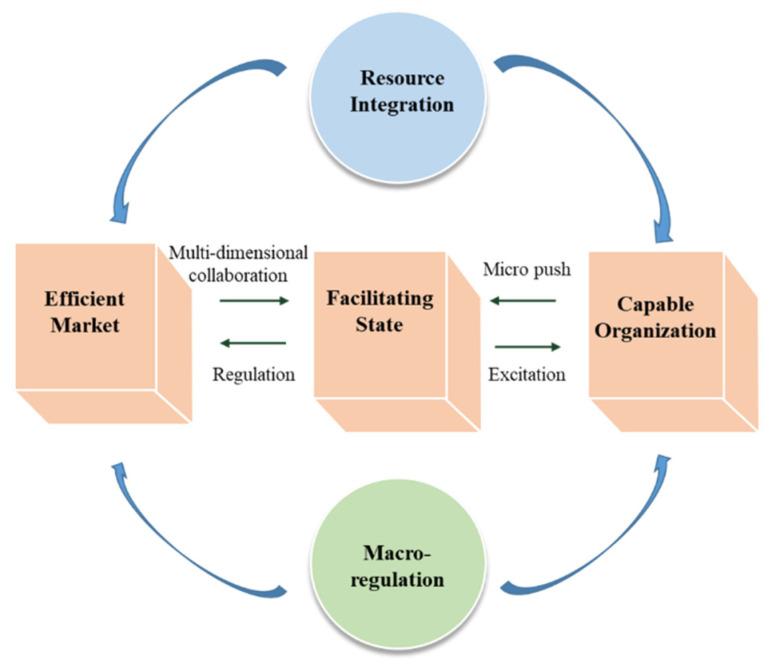
“Three Elements” analytical frameworks of new structural economics.

The facilitating state implements macro-regulation in the economy, and an efficient market promotes the regular operation of the economy. A well-functioning market system can provide adequate support for the facilitating state. However, when the market fails, the government needs to intervene and regulate according to specific conditions, starting with policy determination to maintain economic order [10]; for example, John Y. Campbell pointed out that structuring mortgages can help stabilize the macroeconomic [11]. The enterprises are the micro-subjects that constitute the market, the main force that promotes the effective market and the key to economic development. Social organizations are vital forces in response to crises. Enterprises provide jobs to society while maintaining market order; social organizations are rooted in the private sector, and can more directly perceive personal needs and supplement government actions that are slow and imperfect. Therefore, based on “efficient market and facilitating state”, this article includes “capable organization” as a new element to more fully explain the driving force for the success of pandemic prevention and control. Based on the effective promotion of micro-organizations, the government organizes resource integration and macro-controls the market, thereby achieving a dynamic balance between economic vitality and pandemic prevention control. This mechanism is reflected in the Wuhan city closure, material deployment and inter-provincial health resource coordination. South Korea’s rapid testing technology promotion and telemedicine, and Japan’s public health emergency management system’s synergistic effect also reflect the system’s superiority. Of course, under the norms of market-oriented operation, the government also needs to give effective incentives to various organizations such as enterprises. Only in this way can the micro-kinetic energy of all kinds of organizations be sustainable. The virtuous circle of the system can only be achieved through the facilitating state, efficient market and capable organization (Figure 1).

In this fight against the pandemic, the governments of China, Japan and South Korea have played a significant role in leadership and coordination. Through the integration of various resources, the governments of three countries, on one hand, maximize the efficiency of resource allocation, promote the stability of the market economy and maintain the regular operation of the country; on the other hand, they guide various organizations to exercise their responsibilities to meet the anti-COVID-19 pandemic needs to the greatest extent. It is undeniable that the reason for the success of China, Japan and South Korea in fighting the pandemic is the expected results of the government’s use of efficient market to allocate resources and guide various organizations to complete their missions.

Like fighting the pandemic, China’s economic development experience is unique, and this experience has broken the Washington consensus. In addition, both Japan and South Korea began to develop after World War II and did not follow neoliberalism’s development criteria in economic development. Can the economic construction of developing countries take the path of neoliberalism according to the Washington Consensus? Such questions have been asked for a long time. The unique development experience of China, Japan and South Korea has surpassed the Washington Consensus and embarked on a development path that suits their national conditions. Keun Lee refined the development experience of three countries into the “Beijing-Seoul-Tokyo Consensus” [12]. The consensus mentioned that the common denominator of the development experience of the three East Asian countries lies in the combination of facilitating state, efficient market and capable organization. Like economic development, the anti-COVID-19 pandemic process of China, Japan and South Korea during the COVID-19 pandemic has many things in common, which fully reflects the critical role played by the organic combination of the government, market, and organization in normal economic development and stabilizing social order. This research will combine the “Beijing-Seoul-Tokyo Consensus” and use the “Three Elements” analytical frameworks.

## 3. The Embodiment of Facilitating State

The governments and non-governmental organizations of China, Japan and South Korea donated materials to each other when it was most difficult to fight the pandemic, shared anti-COVID-19 pandemic experience, and exchanged medical information. The respective health, science and technology, and commerce departments of the three parties have strengthened communication, enhanced the sharing and exchange of pandemic information, cooperated in drug vaccine research and development, and coordinated the import and export of medical materials. At the special foreign ministers’ meeting on the COVID-19 pandemic held by China, Japan and South Korea on 20 March 2020, the three parties agreed to discuss the strengthening joint prevention and control, and to explore the development of mutually linked travel pandemic prevention and control guidelines to reduce the probability of a cross-border spread of the pandemic, reduce the impact of the pandemic on economic and trade cooperation and personnel exchanges and stabilize the supply chains of the three countries’ industrial chains [13]. On 15 May 2020, the health ministers of China, Japan and South Korea held a video conference. They issued the “Joint Statement on the Special Video Conference of the 2019 New Coronavirus Disease Pandemic Tripartite Health Ministers Meeting” to strengthen the construction of China, Japan and South Korea information sharing mechanism and deepen the exchange and cooperation of technical institutions [14].

To smoothly carry out the anti-COVID-19 pandemic cooperation between the three countries, we can see from the existing anti-COVID-19 pandemic results that China, Japan and South Korea’s respective response measures in the pandemic are a prerequisite for the three countries to strengthen their anti-COVID-19 pandemic cooperation further. The remarkable achievements of the three countries’ anti-COVID-19 pandemic efforts were promoted by the joint efforts of governments, markets and organizations, and common ground can be found in the anti-COVID-19 pandemic experience: a complete emergency management system for public health emergencies, emphasis on legislation first, clear division of labor, and the system framework and laws are different according to the national conditions of each country; the market also plays a decisive role in the allocation of resources, and works to ensure that information is open and transparent, countries combine the actual situations of government agencies to carry out the most efficient resource allocation; the deployment of medical supplies and funds, enterprises and social organizations are also fully involved in the fight against the pandemic, and there are many differences due to the characteristics of national development. This research explores the success factors of joint anti-pandemic cooperation by analyzing the respective responsibilities of the governments, markets and organizations of China, Japan and South Korea in fighting the pandemic.

### 3.1. The Emergency Management System for Public Health Emergencies and Relevant Laws

#### 3.1.1. China’s Emergency Management System and Legislative System for Public Health Emergencies

**Figure 2:**
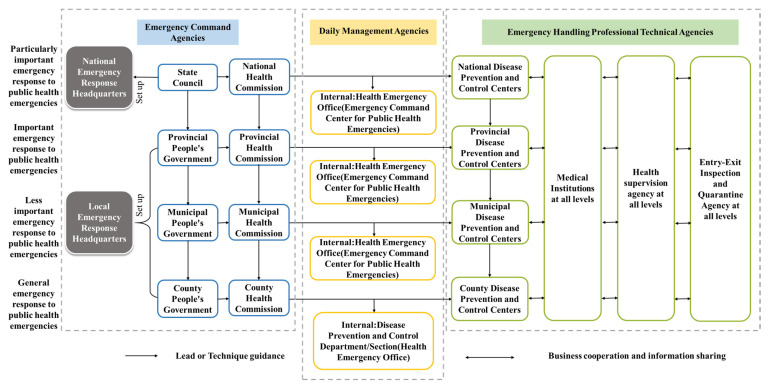
China’s emergency management system.

The formal construction of the emergency management system for public health emergencies in China began with the outbreak of the SARS crisis in 2003. After 17 years of development and evolution, it has been gradually formed in the unified command and leadership of the State Council, the state guiding the localities, and the superior leading the subordinates, top-down and clear levels organizational structure (Figure 2), composed explicitly of emergency command agencies, daily management agencies and emergency handling professional technical agencies. Each agency not only has a business guidance relationship, but also has a linkage mechanism for business cooperation and information sharing, highlighting the principle of “unified leadership, comprehensive coordination, classified management, hierarchical responsibility and territorial management” [15]. State Council and local people’s governments at all levels are the highest administrative leading bodies for the emergency management of public health emergencies in the state and administrative regions. The National Health Commission and the local health commissions are responsible for organizing and coordinating the emergency response to public health emergencies nationwide and in administrative regions. Centers for disease control and prevention at all levels, and medical institutions at all levels, as professional technical institutions for an emergency response to public health emergencies, carry out corresponding emergency response work under the unified command and arrangement of the health administrative department.

The 2003 SARS pandemic gave birth to the “Regulations on Public Health Emergencies”, China’s first administrative regulation to respond to public health emergencies. The “Law on Prevention and Control of Infectious Diseases”, revised in 2004, has played a significant role in preventing and controlling infectious diseases such as avian influenza and influenza A.

At present, China has promulgated 33 anti-pandemic and public health laws, 14 administrative regulations, 5 departmental rules and regulations, and other regulatory documents and judicial interpretations. These public health law sources outline a relatively complete Chinese public health legal framework, including the “Opinions on Punishment of Illegal Crimes Obstructing the Prevention and Control of the Coronavirus Disease Pandemic” issued by the “two high-levels and two ministries” in 2020, which was jointly formulated by the “two high-levels and two ministries” to ensure the smooth development of the pandemic prevention and control work.

#### 3.1.2. Japan’s Public Health Emergency Management System and Legislative System

**Figure 3:**
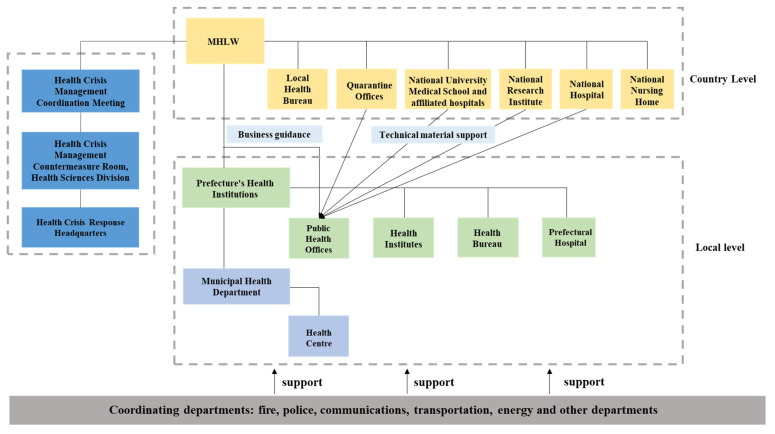
Japan’s public health emergency management system.

Japan’s public health emergency management system consists of two major systems [16] (Figure 3). The central-level public health emergency management system is centered on the Ministry of Health, Labour and Welfare (MHLW), which administers local health bureaus, quarantine offices, national university medical schools and their affiliated hospitals, national research institutes, national hospitals and national nursing homes. MHLW includes a meeting concerning permanent health crisis management and coordination, with a room for health crisis management and countermeasure set up under the meeting. When a public health emergency occurs, a health crisis response headquarters can be temporarily established with the approval of the first-level government to coordinate and manage the emergency.

The local-level public health emergency management system includes prefectures and cities, towns and villages, with Public Health Offices under the prefectures as the center. Public Health Offices are responsible for the prevention and treatment of infectious diseases, and their statutory business includes 14 items, including the prevention of AIDS, tuberculosis, sexually transmitted diseases, infectious diseases and other diseases [17]. Their business is complicated and they have heavy responsibilities. In addition, there are health institutes, health bureaus and prefectural hospitals under the prefecture’s health institutions. Compared to the prefectures, the municipal health department is one level lower than the prefectures, with health centers under it. Therefore, MHLW must provide professional guidance, and the main level’s health agencies provide technical support for them.

Through the cooperation between the health agency and various departments (Table 1), a multi-subject, cross-departmental coordination guarantee system has been established to promptly control the spread of public health emergency crises.

In 1897, Japan implemented legislation to manage infectious diseases and formulated the “Infectious Disease Prevention Law”, which has been revised several times based on disease epidemiology and government implementation. After the spread of H1N1 influenza A, Japan issued a special law titled “New Influenza Countermeasures Special Measures Act” in 2012 [18]. This helps to constitute Japan’s complete public health emergency management legal system with a series of previous policy documents, such as the “Prevention of Infectious Diseases and Medical Treatment for Patients Act”, “Basic Guidelines for Health Crisis Management”, and “Guidelines for Local Health Crisis Management”. In response to the rapid spread of the COVID-19 in Japan, on 13 March 2020, the Japanese Senate voted to pass the amendment to the “New Type of Influenza and Other Special Measures Act”. In strict accordance with the public health emergency management legal system, the government has established affiliated institutions, such as the Health Crisis Management Coordination Conference and the Cabinet Crisis Management Center, to take corresponding countermeasures and formulate action plans for public health emergency management.

#### 3.1.3. Korea’s Infectious Disease Crisis Management System and Legislative System

**Figure 4:**
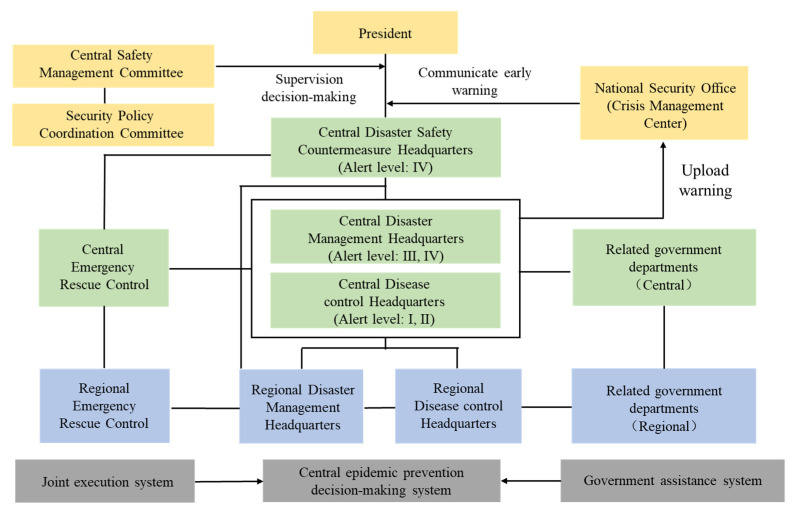
Korea’s infectious disease crisis management system.

The Korean infectious disease crisis management system mainly refers to the institutional setting of the Korean government organization system that integrates disaster safety management and infectious disease prevention and control functions, which is equivalent to the “One Plan, Three Systems” of China’s emergency management system. Following the “Basic Law on Disaster and Safety Management”, the highest guideline for managing various crisis events, and the “Infectious Disease Risk Management Standard Manual” revised under it, South Korea has stipulated the decision-making and implementation system for infectious disease crisis management. The functions of the system including crisis monitoring of disease events, assessing and releasing warning levels of infectious disease crisis, establishing staged pandemic prevention measures and post-disaster recovery management of public health events. With the continuous updating of policies and systems, South Korea has formed a central-local infectious disease crisis management system interconnected from top to bottom and intersected in a three-dimensional way. In other words, when an infectious disease crisis occurs, the central and local governments jointly carry out coordinated emergency management (Figure 4). From a horizontal perspective, South Korea’s infectious disease crisis management system includes the joint execution system of the military, fire, medical and social organizations; a central pandemic prevention decision-making system and a government assistance system. From the longitudinal view, the central system is the baton, with the Central Safety Management Committee chaired by the Prime Minister as the top leadership, the emergency management headquarters—Central Disaster Safety Countermeasure Headquarters, the main front—the Ministry of Health and Welfare, Control Tower—Korea Centers for Disease Control (KCDC), responsible for reviewing designated policies, commanding and coordinating local fire protection, military, medical and other matters. The local system is responsible for epidemiological investigations, laboratory testing, crisis site management, human resources and facility management. The specific work conforms to the principle of vertical management from central to local.

The South Korean government was comparatively slow to make legislation on a public health emergency response. The outbreak of atypical pneumonia (SARS) in 2003 made South Korea realize that the infectious disease crisis was increasing and began to attach importance to the management of infectious disease incidents. However, before 2015, emergency management was still dominated by natural disasters and large-scale accidents, and only a few laws were promulgated for infectious disease crisis management, such as the “Infectious Disease Prevention and Control Law” promulgated in 2010. After the Middle East Respiratory Syndrome (MERS) broke out in 2015, South Korea’s government successively implemented “Increase the budget for disease prevention and research and development” and revised the emergency management of public health emergencies, built an infectious disease prevention system, and formulated the “Respiratory Tract Infectious Disease Pandemic Response”, “Infectious Disease Risk Management Standard Manual” and “Public Health Risk Communication Operation Standard” [19].

After the outbreak of the COVID-19 pandemic, South Korea’s government further revised and improved relevant laws to ensure that there are laws to follow for pandemic prevention and control. In January 2020, the Ministry of Health and Welfare changed the original 1–5 group management of infectious diseases to 1–4 level management. Among them, the most severe first-degree infectious disease is a bioterrorism infectious disease with a high fatality rate, a high possibility of causing group infection and requires harmful pressure isolation. The Department of Disease Management has designated COVID-19 as a first-degree infectious disease. In addition, in response to the problematic situation, South Korea convened the National Assembly on 26 February 2020 and passed the “Infectious Disease Prevention and Management Law Amendment”, “Quarantine Law Amendment” and “Medical Law Amendment”. Provisions for fines and penalties for refusal, obstruction or avoidance of quarantine and isolation have been added, the amendment also stipulates that the Ministry of Health and Welfare may prohibit the export or carrying of medical or non-medical supplies and other materials in the event of a first-level pandemic infectious disease in order to prevent and respond to the price increase or insufficient supply of pandemic prevention and treatment materials—in the case of COVID-19, masks and disinfectants apply to this regulation. When the warning level is raised to “Attention” or higher, children and the elderly who use public welfare facilities and other susceptible people can get mask support [20].

### 3.2. Anti-COVID-19 Pandemic Method and Implementation

#### 3.2.1. “Prevention, Control, and treatment” Entities Have a Clear Division of Labor and Responsibilities in China

After the outbreak of the COVID-19 pandemic, the central level unified command and deployment, the State Council’s joint prevention and control mechanism for the COVID-19 pandemic and the local emergency headquarters established various working groups [21]. For instance, the scientific research team, comprehensive team, works for steering group and medical supplies guarantee team; quickly organized and coordinated the center for disease control and prevention and medical institutions. The core response work, such as epidemiological investigations, pandemic treatment, was carried out and formed a unified leadership and comprehensive coordination of the “national participation” anti-COVID-19 pandemic pattern.

The core response system for public health emergencies is the main body that implements the “prevention”, “control” and “treatment” of emergency medical care. Under the leadership of the Health Commission and other departments, it is composed of the disease prevention and control agencies and the primary medical institutions, minor isolation life centers (such as mobile cabin hospitals) and critical care centers (comprehensive medical institutions, infectious disease hospitals) are specifically responsible. First, the disease control and prevention center collaborates with various professional organizations and popularizes science work. They are responsible for the daily monitoring of the pandemic, undertaking the epidemiological investigation of the pandemic, nucleic acid detection and sampling, setting up a team of experts to conduct research pandemic prevention and control countermeasures, carrying out scientific research work such as pathology and toxicology and emergency science education for the public. Answering key questions like “Infectiousness during the incubation period of the pandemic, and the route of transmission”, “Clinical features and manifestations of the pandemic”, “How to carry out self-protection”, “How to deal with suspected cases” through multiple channels such as popular science webpages and expert interviews [22], raises the overall level of awareness of the pandemic. Second, primary hospitals or clinics do well in community work. The National Health Commission promulgated the “Notice on Further Doing a Good Job in the Prevention and Control of Coronavirus Disease Infection in Primary Medical and Health Institutions” and other policies to guide the grassroots in pre-inspection and triage, key personnel investigation, home isolation management, technical guidance for pandemic prevention, and the development of community grid management and other aspects of work. It is responsible for the health follow-up of discharged patients, and psychological assistance for community people in the pandemic. Third, the mild isolation life center (mobile cabin hospitals) and the critical care center (comprehensive medical institutions, infectious disease hospitals) are responsible for classifying and treating the confirmed population. In order to maximize the effectiveness of emergency medical resources and ensure that patients receive appropriate care, diagnosed patients are classified and treated according to the severity of the disease, and mild and severe patients are distinguished. All confirmed patients are included in the emergency medical system. Daily health care inspections are provided, such as nucleic acid testing and blood routine tests for mild patients. The progress of the disease is monitored, and patients with severe illness tendency are transferred to the critical care center in time; “centralized patients, centralized experts, centralized resources, and centralized treatment” for critically ill patients is implemented in principle. A professional critical care medical team composed of respiratory, infectious diseases, and critical care medicine departments should be established to expand the critical care capacity, and equip corresponding professional teams to support tracheal intubation, extracorporeal membrane oxygenation (ECMO) and other medical technologies, maximizing the utility of medical resources.

#### 3.2.2. Japan’s Emergency Medical Service System Implements Anti-Pandemic Throughout the Process

**Figure 5:**
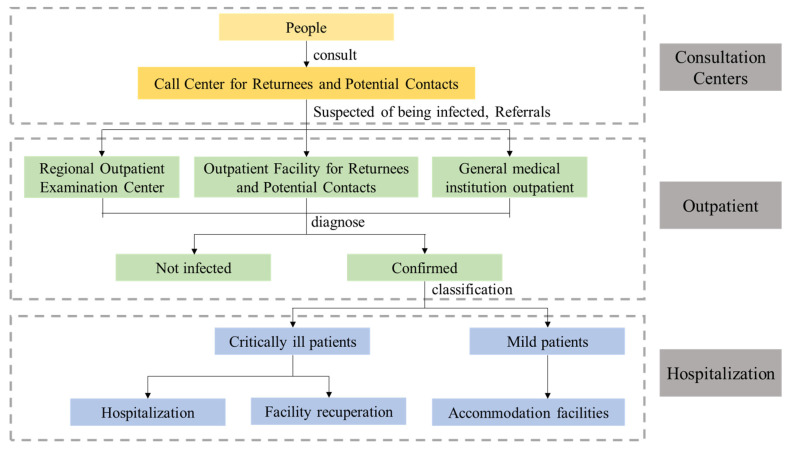
Japan’s emergency medical service system.

Japan has established an emergency medical service system integrating “prevention, control, and treatment”, the specific process of which is shown in Figure 5. Firstly, all prefectures and counties have set up consultation centers for returnees and contacts, open to people who are worried about being infected, to prevent people who are worried about being infected from going to medical institutions for treatment. This is because the Japanese government is concerned that people who are infected will go to medical institutions for treatment, having contact with doctors and patients which will increase the risk of infection. Secondly, outpatient clinics for returnees and contacts, and regional outpatient examination centers are newly established by MHLW and the prefectures. When the above institutions cannot afford medical services, the prefectures will expand the outpatient diagnosis and treatment scope upon consultation with MHLW, which is mainly responsible for screening suspected persons. Thirdly, the MHLW and the prefectures focus on the hospitalization of severe cases. Asymptomatic patients and mild patients are isolated at home or recuperated in accommodation facilities such as hotels to prevent infections within the family and cope with sudden changes in symptoms. In areas where accommodation facilities are fully secured, patients with mild illnesses are based in residential care [23].

#### 3.2.3. Handling and Classified Treatment of Overseas Imported Cases in South Korea

The KCDC requires those who have been to the South China Seafood Market in Wuhan to report to KCDC whenever they develop fever and respiratory symptoms. At the same time, close cooperation with Chinese health agencies and the World Health Organization was initiated to continue to collect information on the pandemic and conduct risk assessments. As the number of suspected and confirmed patients coming to Korea continues to increase, South Korea’s Central Disaster Safety Countermeasures Headquarters announced the implementation of “special entry procedures” for passengers coming from mainland China, which requires all entrants to take their temperature at the airport and fill out a particular quarantine declaration form and health status survey form. All entrants into South Korea must confirm their contact information and address before entering. With the outbreak of COVID-19 pandemic on a global scale, the scope of application of this measure is being gradually expanded to all countries and regions, and the most significant limitation is to control the inflow of overseas cases at airports.

With the rapid increase in the number of confirmed patients and isolated people, South Korea’s medical resources were seriously inadequate and confirmed patients could not be admitted to the hospital in time. In order to cooperate with the strategy of “hierarchical diagnosis and treatment” [24], South Korea converted idle places such as the Central Education and Research Institute, employee dormitories and training centers into “Life Treatment Centers” to isolate and treat patients with mild illnesses. During treatment at the life treatment center, medical staffs need to monitor patients more than twice a day. When the symptoms worsen, patients are quickly transferred to the medical institution; when relieved, they can leave the life treatment center according to the isolation release standard. The life treatment centers in South Korea played the role of China’s “mobile cabin hospital” and played an essential role in curbing the spread of the pandemic to the community. With the improvement of the treatment capacity of life treatment centers, the rapid spread of the pandemic was gradually contained.

## 4. The Embodiment of Efficient Market

The question of promoting market effectiveness in the fight against the pandemic is mainly reflected in two aspects. One is whether the information is complete. Information is one of the most important elements of the market. With sufficient information, it is easier to promote the market to allocate resources better. Governments of various countries actively promote the transparency and timely disclosure of anti-pandemic information. Complete information can play a good role in the market, stabilize consumer and investor sentiments, and stabilize the economy that may be hit hard by the pandemic. Medical resources and emergency funds play an equally direct role in ensuring an efficient market. The second is whether the materials are sufficient. After the outbreak, due to the surge in demand for supplies and market failures, governments of various countries used legal or political means to compel the necessary deployment of medical supplies in pandemic areas or across the country to ensure that supplies are given priority to meet the areas at greatest need to a certain extent, maintaining the relative stability of the market.

### 4.1. Information Disclosure

#### 4.1.1. China Discloses Information Timely about the Pandemic

In combating the COVID-19 pandemic, China has made “full knowledge of the public” a prerequisite for benign communication and interaction between the government and the public. It will timely report on the progress of the fight against the pandemic in various places through press conferences and other forms, publish pandemic case data and organize authoritative experts to pass it. Various channels such as press conferences, media interviews and the Internet release authoritative opinions and professional suggestions on scientific prevention and treatment, and promote the popularization of public protection measures such as wearing masks, frequent hand washing and frequent ventilation, effectively reducing public panic and promoting public awareness of COVID-19. The correct understanding has promoted the conscious participation of the public and enterprises in preventing and controlling the pandemic.

#### 4.1.2. Japan’s High Level of Information Technology to Fight against COVID-19

In the fight against COVID-19, to make medical information transparent and visual, MHLW cooperated with the Cabinet Secretariat’s IT Office to establish an Information and Communication Basic Center to unify the information and communications of approximately 8000 hospitals with 20 or more beds across the country. The operation status, hospital beds and medical staff, the safety status of medical machines (ventilators, etc.) and medical supplies (masks and protective clothing, etc.) can effectively guarantee the precise supply of masks and other materials and precise arrangements for patient transportation.

**Figure 6:**
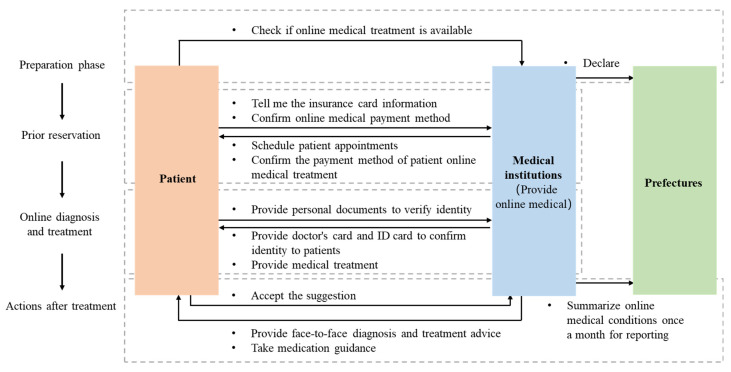
The process of people using the online diagnosis and treatment system during the Japanese pandemic.

Aside from establishing an infrastructure center of information and communication, Japan has also launched an online diagnosis and treatment system developed by MHLW that uses telephones and other communication devices including initial consultations from the perspective of preventing the diagnosis of new coronavirus pneumonia by both the people concerned about infection and medical practitioners (online diagnosis and treatment here refers to the diagnosis and treatment behaviors of doctors using information and communication equipment to diagnose and observe patients, and to communicate diagnosis results and prescriptions). During the pandemic, the operation of the online diagnosis and treatment system is coordinated and managed by the prefectures and counties (Figure 6) [25,26].

#### 4.1.3. National Disaster Prevention and Safety Network of South Korea

Information sharing is an essential foundation for multi-regional and multi-departmental linkage. The disclosure and exchange of various types of information have promoted the rapid collaboration of the Korean public and various agencies to fight the pandemic. On one hand, South Korea attaches great importance to information interaction between social organizations and the public. The Central Disease Control Headquarters publishes pandemic information twice a day, fully respecting the people’s right to know. On the other hand, the national disaster prevention and safety network of South Korea is a unified network based on network technology, which combines the wireless communication networks of relevant agencies such as the ministry of public administration and security, army, fire, medical institutions and social organizations. By combining government and social organizations, the network can realize the real-time exchange of crisis information, share and support the roles of institutions, concentrate resources to coordinate all kinds of needs in emergency management and achieve maximum resource sharing.

### 4.2. Allocation and Guarantee of Medical Supplies

#### 4.2.1. China’s Centralized Allocation of National Emergency Medical Resources

During the fight against the COVID-19 pandemic, China gave full play to its system advantages of “concentrating power to do major events” and coordinated national emergency medical resources deployment. In the early pandemic, when Wuhan medical staff and materials were in short supply, it adopted the method of “counterpart assistance” to organize the whole country. Medical staff rushed to help, the organization of production in terms of materials was optimized, the emergency supply of medical materials and daily necessities was continuously strengthened and medical enterprises overcame difficulties, such as insufficient workers returning to work, and resumed medical supplies production as quickly as possible, laying a material foundation for fighting the pandemic.

In order to coordinate emergency medical rescue forces, the State Council’s joint prevention and control mechanism and local emergency command headquarters made timely decisions to build, expand and renovate the isolation wards of medical institutions and infectious disease hospitals by the development of the pandemic, activating the emergency requisition mechanism for public places, and building mild isolation living centers (such as mobile cabin hospitals) equipped with the corresponding infrastructure to comprehensively improve the treatment capabilities of the emergency medical system.

#### 4.2.2. Japan’s Long-Term Reserve Emergency Fund Guarantees Supplies

Japan’s public health emergency funding sources are relatively comprehensive [27], mainly from the following sources: (1) Public health emergency budget. In response to public health events such as influenza viruses and international infectious diseases, the Central Cabinet Office allocates a fixed budget of 80 million yuan each year. (2) Disaster relief fund. The Basic Law on Disaster Countermeasures stipulates the accumulation of disaster relief funds of local governments, which must accumulate five-thousandths of the local ordinary tax amount for the first three years of the current year and exceed 5 million yuan each year. (3) A special reserve fee was set up. Japan allocated 10.3 billion yuan from the reserve funds to deal with the COVID-19 pandemic, mainly supporting face masks and strengthening border surveillance. It is precisely because of the multi-channel emergency fund guarantee that subsidies ranging from 100,000 yuan or 200,000 yuan per person are available for those who work in key medical institutions designated by prefectures and medical practitioners and employees working in designated medical institutions for infectious diseases and other medical institutions that are allocated hospitalization for the COVID-19 pandemic [28] Through the distribution of subsidies, the shortage of human medical resources has been alleviated to a certain extent. After the COVID-19 outbreak, Japan Congress immediately included the new coronary pneumonia as a disease under the Infectious Diseases Act, and all patients received free treatment [29].

#### 4.2.3. South Korea’s Financial Compensation for the Pandemic Area to Ensure Supplies

The Central Disaster Safety Countermeasure Headquarters designated the worst-hit areas such as Daegu and Gyeongsangbuk-do as “special disaster zones”. In order to help the regions mentioned above overcome the crisis as soon as possible, the central government allocates funds to bear 50% of all disaster-stricken reconstruction expenses, including pandemic prevention management expenses, residents’ living and settlement expenses, and relief funds for the dead and injured. The South Korean government has also adopted various methods to provide services and support to people who are actively participating in pandemic prevention. For hospitalized or quarantined residents without income, the government provides 5 million face masks to designated public agencies which they can sell at preferential prices. The South Korean government also provides non-discriminatory treatment for the Chinese and overseas students living in South Korea; anyone diagnosed in South Korea will receive free treatment and isolation subsidies.

## 5. The Embodiment of the Capable Organization

After the governments of various countries have concentrated their efforts to fight the pandemic, they have issued instructions to various organizations. From the instructions to the prompt response of each organization, this reflects the organization’s enthusiasm and contribution in the fight against the pandemic. In addition, companies, social organizations, and grassroots institutions in various countries have also responded quickly to the pandemic. Enterprises and social organizations from various countries actively assumed their social responsibilities during the pandemic, and devoted themselves to the fight against the pandemic, demonstrating the ongoing family and country focus of the Han cultural circle.

### 5.1. Active Actions of Chinese Enterprises and Grassroots Organizations

#### 5.1.1. Companies and the Public Highly Involved in the Fight against the Pandemic

During the fight against the COVID-19 pandemic, the Chinese people actively responded to the government’s prevention and control deployment, consciously promoting the formation of a national anti-COVID-19 pandemic that includes the government, enterprises, social organizations, grassroots communities, volunteer groups, families and individuals. Measures such as long-term “closure of the city”, community access management and national home isolation have been strictly implemented. Four million urban and rural community workers across the country have actively participated in “grid management”. During the pandemic, Wuhan’s community forces launched a carpet search for the community grid investigation. The investigation of 10.59 million people in Wuhan was completed within three days, from 17 to 19 February. One thousand four hundred ninety-nine cases of severely ill patients were detected and the first barrier for pandemic prevention, control and management was constructed to reduce the pandemic effectively. The infection rate has laid a solid social foundation.

Aside from donating money and materials, Chinese companies take advantage of their expertise to actively participate in the fight. Traditional production companies have expanded their production capacity to ensure the supply of medical supplies such as masks and medicines. Internet platform companies and logistics companies such as search, social networking, and e-commerce and food delivery have effectively assisted the anti-COVID-19 pandemic process with their infrastructure and business advantages. Online pandemic maps such as Dingxiangyuan website provide a convenient window for the general public to understand the dynamics of the pandemic. China Construction Third Engineering Bureau is the main contractor, coordinating various participating entities to build the Huoshenshan Hospital, providing an infrastructure guarantee for pandemic treatment. China Mobile, Tencent, Baidu, etc., successively provided real-time population migration data to provide data reference for different regions and assist in pandemic management. By taking full advantage of their business, the participation of various types of enterprises in the fight against the pandemic has effectively improved China’s health emergency management modernization.

#### 5.1.2. Mobilize Grassroots Forces for Investigation

During the prevention and control of the COVID-19 pandemic, we attached great importance to “source prevention and control”, with the staff of primary medical institutions as the technical support under the network, and fully encouraged grassroots community forces and party organizations to participate in the “grid management” work, including personnel inspection and technical guidance for pandemic prevention, etc., with communities and village committees as units, implementing the policy of “early detection, early reporting, early isolation, and early treatment”. Grassroots organizations urge residents to conduct self-inspection and self-report through propaganda, and at the same time carry out a carpet ceremony at home. Investigation and adjustment of the “external and internal prevention” strategy based on changes in the pandemic situation, screening of confirmed patients, suspected patients, close contacts and other groups, effectively moves forward the barriers of pandemic prevention and control.

### 5.2. Japanese Companies and Social Organizations Respond in an Orderly Manner

#### 5.2.1. Enterprises Implement Emergency Management Regulations

During the fight against the pandemic, Japanese companies focused on two aspects: (1) Organizing and establishing countermeasure headquarters. Enterprises’ response to the COVID-19 pandemic is divided into three progressive stages. The first stage is to collect and analyze information. When the pandemic worsens and escalates, simply collecting information cannot play a significant role in fighting the pandemic, and it needs to enter the next stage; the second stage is to hold a countermeasure meeting. Companies need to hold a countermeasure meeting, and the corporate crisis management committee will discuss and negotiate whether to set up a countermeasure headquarters to deal with the pandemic following emergency management regulations; the third stage is to formally establish a countermeasure headquarters, when the pandemic continues to worsen and develop, the company should establish a countermeasure headquarters, negotiating and communicating with the staff of the government, other agencies and enterprises. (2) The company’s own specific measures to combat COVID-19, including encouraging employees to work from home, prohibiting business trips to pandemic areas, implementing a business travel isolation system, avoiding crowded vehicles, prohibiting handshake and hug etiquette, encouraging employees to wear masks daily, comprehensive upgrade disinfection measures, strengthening daily hygiene management and checking the reserves of disaster prevention supplies are detailed in Table 2 below.

#### 5.2.2. Social Organizations Receive Guidance and Emergency Response

In Japan, social organizations are called Non-Profit Organizations (NPO) or Non-Governmental Organizations (NGO). Social organizations developed slowly in the early days of Japan. As their number increases, the types of activities of Japanese social organizations were gradually enriched, including health promotion, medical treatment and welfare, disaster relief, community safety activities, etc. In fighting against the pandemic, social organizations were led and managed in a unified manner to maximize their effectiveness.

For example, the Disaster Medical Assistance Team (DMAT) is a member of many social organizations in Japan. A medical team starts activities during the emergency period (within approximately 48 h) after an emergency occurs and receives specialized training. Under the standard system, MHLW formulates activities for DMAT, and at the same time, maintains and improves the quality of DMAT through the implementation of standardized training, training, certification and registration of DMAT staff. In the COVID-19 pandemic, DMAT accepts the leadership of the Ministry of Health, Labour and Welfare and prefectures, and is mainly responsible for activities such as area-wide medical delivery, hospital support, regional medical delivery and on-site activities. It also needs to be responsible for information collection and other business.

### 5.3. Close Cooperation between the South Korean Government and Organizations

#### 5.3.1. Companies Respond to Government Testing Needs

In the face of the infectious disease crisis, the South Korean government has fully mobilized all available medical resources to provide guarantees for providing the most significant possible diagnosis and treatment opportunities and shortening treatment time. KCDC, MHLW, and the Ministry of Food and Drug Safety have issued medical Emergency Use Authorizations (EUA) to some companies. In the case of COVID-19, four biotech companies including KOGENE, were issued emergency authorization for the use of the COVID-19 diagnostic reagent, enabling the rapid approval of the test kits, reaching the production of 15,000 kits per week, and testing volume supporting more than 400,000 person-times per week, greatly supporting the prevention and control of the pandemic in South Korea. At the same time, the government has also delegated the power of virus detection to primary private medical institutions, so that the technical capabilities of enterprises and private hospitals have been fully released.

#### 5.3.2. Multi-Party Linkage of Social Organizations

Social organizations are essential members of the Korean National Disaster Prevention and Safety Network, including various social organizations or groups in South Korea. Through this network, social organizations can achieve multi-party linkages between their primary responsibilities (Table 3) and government departments, medical departments, education departments and other institutions to cooperate to form a joint force in the rescue and assistance system [30].

## 6. The Synergistic Characteristics of the Successful Fight Against the Pandemic

To summarize the reasons for the success of China, Japan, and South Korea in the fight against the pandemic: Firstly, each country has a relatively complete public health emergency management system. After the outbreak of the COVID-19 pandemic, the government quickly responded and issued instructions to trigger emergency plans promptly and adjust the original laws to make them more targeted to the actual occurrence of this pandemic. Secondly, the three countries have reached a consensus on information and technical exchange and established a joint prevention and control mechanism to maximize information efficiency. Thirdly, the limited medical resources were taken into account, and graded treatment for people with mild, severe and asymptomatic infections was carried out. Fourthly, the government has implemented centralized deployment of scarce funds, medical resources and human resources to avoid delays in prevention and control efficiency due to asymmetry between resources and demand. Fifthly, government departments promptly disclose information about the pandemic to the public, popularizing knowledge about the prevention and control of COVID-19, and stabilizing public sentiment.

Organizations in China, Japan and South Korea have all made significant contributions to preventing the spread of the pandemic. On one hand, enterprises actively responded to the government’s call to produce medical supplies to supplement the surge in demand; on the other hand, the social organizations’ scope of responsibilities and capabilities complement those of government departments.

In addition to the reasons mentioned above, China, Japan and South Korea have successfully combated the pandemic, with the countries also forming some unique anti-COVID-19 pandemic mechanisms based on their national conditions.

### 6.1. The Joint Efforts of Chinese Local Governments and Professional Institutions

An important manifestation of China’s facilitating state is strong execution. The outbreak of the pandemic coincided with the Spring Festival. In order to avoid the risk of transmission due to crowd movements and gatherings, after the government issued an order to extend the Spring Festival, local governments immediately issued notices of suspension of work and school, and enterprises and schools implemented this. Later, the practice of various countries has proved that the decision to control the flow of people when the pandemic is severe is exceptionally correct.

#### 6.1.1. Organize Counterpart Assistance for Emergency Resources across Provinces and Cities

China adopted the method of “one province and one city” to support the prevention and control of the pandemic in Hubei from various aspects such as medical supplies, living supplies, medical personnel, finances and materials. According to statistics, China allocated these medical resources from the country in a short time. Three hundred forty-four medical teams and 42,342 medical staff rushed to assist Hubei [31], effectively improving the emergency response capacity of Hubei, the hardest hit by the pandemic.

#### 6.1.2. Organize Various Institutions to Conduct Joint Scientific Research

The science and technology department and the National Health Commission and other 12 departments jointly conduct scientific research, focusing on the current five main directions to deploy emergency tasks. The five directions are: optimizing clinical treatment plans and drug screening, detection technologies and products, viral etiology and pandemics and vaccine development. Thanks to a series of safeguard measures and the active promotion of scientific and technological workers, China’s nucleic acid detection reagents need half an hour to confirm the diagnosis from the initial stage of research and development, and it only takes a few minutes to develop more mature detection reagents, saving the time of doctors and patients and dramatically improving the efficiency of clinical diagnosis and treatment.

### 6.2. Japan Launches Online Diagnosis and Treatment, Training Emergency Professionals

#### 6.2.1. Use Online Diagnosis and Treatment Resources to Reduce Doctor-Patient Costs

Starting from a position of preventing both patients and medical practitioners from contracting COVID-19, MHLW of Japan developed an online diagnosis and treatment program during the COVID-19 pandemic, and launched a medical system that uses telephones and other communication devices, including for the first diagnosis. Through online medical treatment, the sharing of domestic high-quality medical resources is realized, the frequent contact between doctors and patients is reduced, the risk of cross-infection is reduced, and the time and social costs for patients to travel back and forth are saved, medical supplies are saved and medical order is stabilized.

When a medical institution receives a patient’s request for medical treatment via telephone or online, it shall report to the counter in the prefecture. In addition, on the homepage of the medical institution’s website, it is necessary to indicate the period of online diagnosis and treatment, the method of appointment, etc. In addition, on the homepage of the medical institution’s website, it is also necessary to indicate the symptoms of online diagnosis and treatment or the situation that requires face-to-face diagnosis and treatment, to nip it in the bud. The patient consults the medical institution that provides online diagnosis and treatment services; according to the doctor’s online judgment, the patient chooses whether to go to the medical institution for diagnosis and treatment, and it is recommended that the patient choose the medical institution closest to home.

When a patient receives an online diagnosis and treatment, if the doctor recommends going to a medical institution for face-to-face treatment, the patient needs to go directly to the medical institution to avoid delay in treatment and spread of the pandemic. However, if the patient is prescribed and wants to receive medicine after receiving the online diagnosis and treatment, they need to notify the nearest pharmacy to collect the medicine; the patient may also need to go to the pharmacy to obtain medication instructions.

#### 6.2.2. Disseminate Emergency Knowledge by Specialized Agencies and Organizations

Japan attaches great importance to the training of professionals in public emergency management. In July 2002, the Japanese government approved the establishment of the “Japan Disaster Prevention Agency”, this organization specializes in training crisis management personnel, who usually conduct investigations and studies on historical disasters and assist the government in raising public awareness of disaster prevention and mitigation, and enhancing relevant skills; participating in volunteer activities when disaster strikes to rescue the victims. Not only that, but educational institutions have also opened departments or majors in crisis management, for instance, Chiba Institute of Science in Japan established the Department of Crisis Management in 2004 [32], dedicated to cultivating professionals with comprehensive crisis response capabilities.

Social organizations also help the government to educate the public about emergency knowledge. The work of non-profit organizations for disaster prevention and relief includes conducting disaster relief training, providing citizens with disaster prevention information and materials, and popularizing disaster prevention knowledge. In addition, the government has specially allocated aid funds to build residential disaster prevention associations in most towns and communities in Japan, and conduct disaster prevention drills for the people with the support of various academic institutions [33].

### 6.3. South Korea’s Pandemic Prevention System Supports and Prevents Market Risks

#### 6.3.1. The Pandemic Prevention Command System Is Operating Well

South Korea’s infectious disease crisis management system responds to the COVID-19 pandemic with the KCDC, other agencies support and assist KCDC. KCDC has demonstrated its expertise and authority as a pandemic prevention “control tower” from publishing information to organizing pandemic prevention. With the increase in the level of early warnings and the higher authority of the pandemic commander, the Central Disaster Safety Countermeasure Headquarters and the Central Disease Control Headquarters are also advancing multi-government cooperation in an orderly manner. Not only did they fail to shake KCDC’s core role in pandemic prevention, but they further strengthened its position. Regardless of the infectious disease crisis stage, KCDC is responsible for the command of specific pandemic prevention work, which effectively improves the response capacity and pandemic prevention effect of the entire health system.

#### 6.3.2. Carry Out Pandemic Prevention Work Strictly in Accordance with the Law

After MERS in 2015, South Korea amended the “Infectious Disease Prevention and Management Act”, which stipulates that during the pandemic period of infectious disease, relevant departments must disclose the patient’s movement route to the whole society, including the means of transportation used, visiting medical institutions and contact persons. Therefore, many information disclosure measures of the South Korean government in this pandemic are legally based. In addition, when the infectious disease crisis management system encounters problems that have no detailed provisions in the law but have a tangible impact on pandemic prevention during the actual pandemic prevention process, the South Korea National Assembly quickly convened to pass several legal amendments to maintain the mandatory and guarantee of government actions. This has further improved the execution and deterrence of pandemic prevention measures.

#### 6.3.3. Issue EUA

In order to meet the needs of later monitoring, South Korea quickly launched the research and development activities of testing reagents. The Ministry of Health and Welfare has issued EUA to relevant biotech companies, and has submerged virus testing in dozens of private medical institutions to prepare for the large-scale spread of the pandemic. The EUA system refers to the authorization of specific medical supplies (drugs, biological products and medical equipment) to be used for the diagnosis, treatment and prevention of diseases in the event of serious life-threatening diseases and safety emergencies [19], significantly shortening the time required for drugs and reagents to be put on the market. The introduction of EUA has saved the relevant approval process at least eight months, enabling South Korea to have strong virus detection capabilities before the emergence of a large-scale pandemic.

#### 6.3.4. Market Operations to Prevent and Control Financial Risks

With the rapid spread of the COVID-19 pandemic, the South Korean stock market was hit hard, triggering the circuit breaker mechanism many times. The Korea Composite Stock Price Index fell more than 30% in one month. During the pandemic, the Korean Won depreciated sharply, a record low in 11 years. That day, President Moon Jae-in presided over the first emergency economic meeting to respond to the COVID-19 pandemic and announced that large-scale financial relief measures would be launched, with a total amount of up to 50 trillion Won (approximately 277 billion Yuan), stabilizing the livelihood of the people affected by the pandemic. On the same day, the Bank of Korea and the US Federal Reserve signed a six-month currency swap agreement with a scale of 60 billion U.S. dollars. This scale is approximately twice that of the 2008 financial crisis. The Bank of Korea stated that it would continue to cooperate with the central banks of major countries and make every effort to stabilize the financial market [34].

## 7. Conclusions

The prevention and control of the COVID-19 pandemic is a challenge to the governance capabilities and to the prevention and control capabilities of all of society. It needs to be based on an efficient and reasonable coordination pattern. This coordination also includes the abandonment of regionalism by the country’s main body, assistance and the active alliance of various domestic stakeholders to form a joint force. Taken together, China, Japan and South Korea have shown satisfactory anti-pandemic performance in the world, which is closely related to the various policies implemented by the three countries and the active interaction of various organizations. In the logical framework of new structural economics, with the dynamic development of the economic system, the factor endowment and its structure also present a dynamic evolutionary spiral change. The comparative advantage determined by factor endowments and structure is only manifested as a potential comparative advantage. This comparative advantage can become the reality of industrial development and ultimately improve social welfare, depending on the government’s reasonable policy matching and coordination. Therefore, this article starts from the new structural economics, beginning at the three-dimensional analysis framework of the facilitating state-efficient market-capable organization, and comprehensively analyzing the internal driving force of China, Japan and South Korea’s anti-pandemic success.

First of all, the suddenness of public health incidents often results in unclear roles and confusion among all parties involved, and the management order established between social entities in the past fails [35]. In the COVID-19 pandemic, the governments of the three countries have fully demonstrated their effective coordination capabilities for all social participants. They have played an essential role in managing public health emergencies, based on relatively complete public health emergency management systems and legal systems, China, Japan and South Korea can adjust and supplement at any time and place to build a stable macro environment, supplemented by the division of labor, classified treatment, the entire anti-pandemic process, etc. The specific methods give full play to the government’s policy-making advantages and reflect the efficient operation and responsibility of the facilitating state, but also consistent with the conclusions of Christopher Avery, government regulation and policy formulation are very important to combat the pandemic [36]. At the same time, China, Japan and South Korea have held several joint meetings to maintain coordination on pandemic information sharing, cooperation in protest measures, management of entry and exit personnel, stability of the regional industrial supply chains, and promotion of economic recovery, achieving sharing and cooperation among transnational governments. 

Secondly, information and resources are important market elements. On one hand, the use of technology in emergency management scenarios to promote transparency and timely disclosure of information has received the attention of relevant departments, such as the Korean government’s “Tracking-test-treat” strategy [37], and China’s “health code.” Governments use information tracking and information disclosure systems so that complete information can play a good role in the market, stabilize consumers and investor sentiment, and minimize instability of economies that may be caused by the pandemic [38]. On the other hand, the adequate reserves and deployment of medical resources have effectively reduced market failures caused by the surge in resource demand during the pandemic, the government’s financial subsidies can also compensate to a certain extent for the mismatch of resources caused by the market failure [39].

Finally, under the leadership of the facilitating state, enterprises and social organizations have developed their maximum effectiveness. Generally speaking, companies tend to accumulate limited resources to maximize their benefits, and profit-seeking is prominent [40,41]. However, after the outbreak, enterprises and social organizations took active actions, implemented emergency management regulations, formulated pandemic response measures, and actively responded to testing needs to make commercial benefits and social benefits gradually converge; social organizations linked the government, medical and education and other departments to form a joint force in the rescue assistance system. As one of the microscopic entities that make up the market and social organizations indispensable in response to crises, the two give full play to their initiative and become the leading force in promoting pandemic prevention and control and efficient markets.

In short, through the summary and analysis of the specific measures taken by China, Japan and South Korea to fight the pandemic, the reasons for the success of three countries in the fight against the pandemic are multiple—that is, the organic combination of facilitating state, efficient market and capable organization. Based on active joint and effective promotion of capable organizations, China, Japan and South Korea have maintained their market effectiveness through integration and macro-control of facilitating state, realizing a dual and stable win-win situation for pandemic prevention and control and regional economic governance.

## Figures and Tables

**Table 1:** Inter-departmental cooperation and division of responsibilities during public health emergency management.

Number	Cooperation Department	Cooperation Matters and Division of Responsibilities
1	Physician Association, Medical Institution	Use a wide range of disaster and emergency medical information systems to confirm the community’s access to emergency medical care, and take into account the scale of public health emergencies, cooperate with physician associations and community medical institutions to quickly adjust hospital beds and other medical resources; after consultation with the headquarters, requesting assistance from medical associations and medical institutions, and dispatching rescue teams and other medical personnel to the scene.
2	Police department, fire department	Under the leadership of the prefectural health authority, the Public Health Offices establishes a tripartite cooperation mechanism with the police department and the fire department to sign an advance agreement on cooperation in public health crisis emergency management. After an emergency occurs, the police and the fire department assist in the investigation of the health center Information, find out the cause.
3	Local health research institutes, university institutions and other experimental research institutions	when necessary, Municipal Health Department should inquire about the properties, health effects, treatment methods and collection methods of the causing substances; when the local health research institutes have difficulty in identifying the cause or lack knowledge, it is necessary to request other local health research institutes and universities Institutions and other experimental research institutions provide assistance.
4	Local physician associations, medical institutions, welfare departments, fire departments, education departments	Public Health Offices works with local physician associations, welfare departments, education departments, and medical institutions to provide assistance to vulnerable groups (patients with intractable diseases, mental illnesses, patients who use artificial respirators at home, and home dialysis patients who still need “home medical services” after the crisis. Patients, etc.) provide necessary medical services.
5	Japanese Red Cross	The Japanese Red Cross provides emergency medical services and technical support for pandemic prevention and control.
6	Self-Defense Force	In the event of large-scale disasters such as natural disasters or special disasters such as explosions in hazardous chemical manufacturing plants, prefectures should also consider requesting assistance from the Self-Defense Forces.
7	Experts	When responding to health crises, Public Health Offices needs to use the list of experts prepared at ordinary times to listen to the opinions of experts on necessary countermeasures.

**Table 2:** Japanese companies took specific measures to fight the pandemic.

Pandemic Response	Specific Measure
Encourage employees to work from home	In 2018, Japan successfully passed the “Working Style Reform Association Law” to adjust the working methods of Japanese employees, laying a foundation for Japanese companies to fight against the pandemic. Japanese employees are adapted to work from home.
No business trips to pandemic areas	In the outbreak of the pandemic, compared with ordinary areas, the risk of infection in the pandemic area is higher than that of ordinary areas. Based on the judgment of the severity of the pandemic, Japanese companies strictly approve employees’ applications for business trips in the pandemic area. When the pandemic area is in an uncontrollable state, employees are clearly prohibited from traveling to the pandemic area.
Implement a business trip isolation system	When an employee returns from a business trip from a pandemic area, they should report their business trip to the company and the community where they live, and actively self-quarantine for 14 days. During the quarantine period, pay attention to your own physical condition. When you feel unwell, you need to report to the company. The company will make an appointment for the hospital, and the company will bear a series of medical expenses for the employees.
Avoid riding in crowded vehicles	During the peak period of work, personnel contact cannot be avoided. During the pandemic, companies can appropriately shorten the working hours of employees to avoid the probability of infection caused by employees riding in crowded vehicles.
Etiquette such as shaking hands and hugging is prohibited	During the pandemic, Japanese companies clearly required employees to maintain a safe distance of more than 1 m, and no friendship etiquette such as shaking hands and hugging.
Encourage people to wear masks daily	To avoid cross-infection, Japanese companies encourage employees to wear masks to minimize the infection rate.
Comprehensive upgrade of disinfection measures	During the new pneumonia pandemic, some Japanese companies optimized and upgraded their disinfection measures. For example, provided sufficient hand sanitizer, disinfecting paper towels, alcohol disinfectant; increased the number of disinfection without dead ends every day.
Strengthen daily hygiene management	With a high rate of disasters in Japan, Japanese companies and people have formed a strong sense of emergency and actively strengthened daily health management to take precautions.
Check the stock of disaster prevention supplies	Japanese companies will regularly check the internal reserves of disaster prevention supplies. When the reserves are insufficient, they will be supplemented in time to ensure that they have sufficient material reserves to deal with disasters.

**Table 3:** Korea Disaster Prevention and Safety Network member organizations or groups and their main responsibilities.

	Member Organization or Group	Main Responsibilities
Formal members	Korean Nurse Association	A service organization with nurses as the mainstay to protect national health
	Korean Medical Association	A group of medical experts dedicated to maintaining national health and medical development
	National Volunteer Fire Association	Volunteer firefighting teams that carry out disaster prevention activities and provide on-site support
	Korean Red Cross	Statutory disaster management agency responsible for carrying out relief activities
	Korea Saemaul Undong Center	A non-governmental organization formed by citizens to assist in disaster relief activities
	Korea Rescue Association	On-site support team to protect people’s lives and property in the event of a disaster
	Volunteering Korea	A by-law umbrella organization for Korea voluntary bodies, advocate volunteerism and make efforts to create an enabling environment for volunteering.
	Korea Volunteer Center	Overall management of national volunteer teams or organizations
	Safe Life	Non-profit private organizations that mobilize citizens to respond to various security incidents
	R.O.K. Marine CORPS V.A	Naval volunteer team to provide disaster field support
	The Korean Amateur Radio League	Social groups that provide wireless communication support and network technology guidance
Cooperative members	Community Chest of Korea	Representative private fundraising agency in the field of Korean social welfare
	Disaster Prevention and Safety Management Research Center of Yonsei University	Research on disaster prevention and management strategies
	Korea Facility Safety Corporation	Ensure the safety of public facilities and protect people’s lives and property
	Crisis management theory and practice team	A research team composed of academic researchers and practitioners in the field of crisis management, aiming to put various crisis management theories into practice
	119 Magazine	Issue special issue of disaster safety information and fire prevention
